# Indications and Overuse of Computed Tomography in Minor Head Trauma

**DOI:** 10.5812/ircmj.13067

**Published:** 2014-05-05

**Authors:** Sanaz Zargar Balaye Jame, Reza Majdzadeh, Ali Akbari Sari, Arash Rashidian, Mohammad Arab, Hojjat Rahmani

**Affiliations:** 1 Department of Health Management and Economics, School of Public Health, Tehran University of Medical Sciences, Tehran, IR Iran; 2 Department of Epidemiology and Biostatistics, School of Public Health, Tehran University of Medical Sciences, Tehran, IR Iran; 3 Knowledge Utilization Research Center, Tehran University of Medical Sciences, Tehran, IR Iran; 4 Department of Health Care Management, School of Applied Medical Sciences, Tehran University of Medical Sciences, Tehran, IR Iran

**Keywords:** Tomography, X-Ray Computed, Craniocerebral Trauma, Indication, Abnormal

## Abstract

**Background:**

Computed Tomography (CT) is a useful diagnostic technology, particularly in accident and emergency departments.

**Objectives:**

To identify a comprehensive list of indications for application of CT in patients with minor head trauma (MHT) and to determine appropriateness of its use on the basis of this list.

**Materials and Methods:**

A cross-sectional study was conducted in three Imaging centers in Tehran. A panel of experts developed a list of CT indications for MHT by reviewing documents. A pre-structured checklist was designed and incorporated into a structured form. Four hundred consecutive patients referring to three imaging centers for performing CT due to MHT completed the questionnaire.

**Results:**

Of 400 patients who underwent CT after MHT, 187 (46.8%) patients had Glasgow coma scale (GCS) score of 13 or 14 at two hours post-trauma and 37 (19.8%) of these patients did not have any indication of imaging. In addition, 213 (53.2%) patients had GCS score of 15 out of which 110 (51.6%) patients did not have any indication of imaging. Patients with a GCS score of 15 had a noticeably lower proportion of abnormal CT results in comparison to patients with a GCS score of 13 or 14, (odds ratio, 19.07; 95% confidence interval, 6.74-54.00; and P < 0.001). There was a statistically significant association between abnormal CT results and the presence of indications including vomiting, dangerous mechanism of injury, visible signs of trauma above the clavicles, signs of skull base fracture, and suspected skull fracture (P < 0.001).

**Conclusions:**

On average, about 37% of the patients with MHT referring to the emergency departments had no indication of CT and approximately 86.5% of CT results were normal. Improving this situation can result in a significant saving in health care costs.

## 1. Background

Computed Tomography (CT) is an important diagnostic tool in many emergent conditions; however, it might be overused in many health care systems. This might lead to a significant drain on health care resources as well as increased risk of radiation exposure (1, 2). The growing number of appearing health technologies helps to fulfill both patients’ and physicians’ demands (3). Studying the utilization patterns of diagnostic imaging technologies is important for health care management, especially in developing or underdeveloped countries (4). Medical imaging costs have had an increasing trend all over the world, which might be a result of their overuse (5). There are many methods and criteria for evaluating the appropriateness of health care services (6). Decision principles can not only decline the costs and the number of performed imaging, but also decrease the emergency and radiology wards overcrowding (7).

Nearly, half of the all CT scans performed in the emergency departments are head scans (8, 9). A recent study has shown that 10% reduction in the number of CT scans in patients with minor head trauma (MHT) could lead to an annual saving of more than 20 million dollars (10). It seems that in Iran, a significant proportion of CT scans is performed without any indication. This issue, however, has not been well studied in Iran.

## 2. Objectives

The objectives of this study were to identify a comprehensive list of indications for utilization of CT scan in patients with MHT and to determine the level of appropriate use of this tool in MHT patients based on this list in Iran.

## 3. Materials and Methods

### 3.1. Identifying the List of Indications of the Computed Tomography in Minor Head Trauma

Medline was searched to identify the reports concerning appropriateness criteria, standards, and indications of CT in patients with MHT. Nine relevant documents were identified of which one report provided a comprehensive list of the indications (11). We also added related items that were identified in other reports to complete the list of indications (12-18).

An applied method for head injury classification is dividing head injuries into minor or major groups based on patient’s Glasgow coma scale (GCS) score. Trauma management starts with determination of the patients’ GCS in the initial assessment. This scale is apparently correlated with severity of the damage happening inside the skull; besides, it can be measured with sufficient reliability by the health care providers. Therefore, GCS is a widely accepted measure of severity of neurological trauma. Major head injury includes patients with GCS score of 3 to 12. Minor head injury includes patients with GCS score of 13 to 15 (7).

The list of indications was translated into Farsi and was checked by two physicians. Six specialist residents from the three major specialties, namely emergency medicine, neurosurgery, and neurology who are involved in the ordering of CT for MHT, were assigned to check the structure and content validity of the questionnaire. The preliminary checklist was sent to these residents and based on their comments, we modified the questionnaire. The final list of indications was identified based on the general consensus made on the panel of these six residents. The panel approved nine indications out of the ten indications that were identified by the literature review. Head CT scan in patients with MHT (GCS of 13 to 15) was necessary only when one or more clinical risk factors were observed. Abnormal CT findings included the results that required neurosurgical care (observational or interventional).

### 3.2. Setting, Sampling, and Patient Recruitment for Field Study

The following formula was used for sample size calculation: n = Z^2^ pq/d. The field study was performed in three imaging centers in two large public hospitals and a medium military hospital in Tehran. Then 150 and 100 consecutive patients from each large hospital and the medium hospital, respectively, were selected. Overall, 400 individuals from both genders were recruited from September to December 2012.

The prestructured checklist was used to obtain the information from selected patients through face to face interviews by trained interviewers (emergency department nurses) and patients’ medical records. The inclusion criteria were GCS score of 13-15, age ≥ 3 years, and a MHT during the past 12 hours. Collected data were analyzed using SPSS v. 19 software for Windows (SPSS Inc., Chicago, Illinois). Chi-square and Fisher’s exact tests were used to evaluate statistical significance. We quantified the strength of associations using the odds ratio.

### 3.3. Ethical Concerns

The Institutional Review Board of Tehran University of Medical Sciences approved this study (number: 18741, January 16, 2012). Hospitals administrators agreed with implementing the study at the emergency departments. Verbal permission was taken from patients and their relatives after explaining the importance of the study. Participation in the study was voluntarily and based on the informed consent obtained before completing the questionnaire. We did not perform any intervention in the study and did not make any change in the process of patient diagnosis and treatment.

## 4. Results

Of 400 patients, 228 (57%) respondents were males. The patients’ average age was 36.9 ± 19.6 years. Overall, 262 (65.5%) patients had insurance coverage. Amongst the respondents, 23%, 42%, and 88.8% were housewives, had diploma education level, and lived in urban areas, respectively.

### 4.1. Indications of Computed Tomography in Patients With Minor Head Trauma

Among the study participants, 147 (36.8%) patients had no indication and 253 (63.2%) patients had at least one indication of CT. Approximately, 86.5% of CT findings were normal. From 253 patients that had indications of CT, 180 (45%) and 88 (22%) patients underwent CT due to dangerous mechanism of injury and signs of skull base fracture, respectively (Table 1).

The GCS score of 13 to14 at two hours post-trauma was reported in 187 (46.8%) patients among which 37 (19.8%) patients did not have any other risk factors. GCS score of 15 was reported in 213 (53.2%) patients among which 110 (51.6%) patients did not have any other risk factors. CT reports according to GCS score are shown in Table 2.

In terms of abnormal CT results, there was a major difference between patients with the GCS score of 13 to 14 and those with the GCS score of 15 (odds ratio, 19.07; 95% confidence interval, 6.74-54.00; and P < 0.001).

The correlation between the indications of CT and abnormal findings are shown in Table 3. There was a statistically significant association between abnormal findings in CT and the indications of CT including vomiting, dangerous mechanism of injury, visible signs of trauma above the clavicles, signs of skull base fracture, and suspected skull fracture (P < 0.001). The risk of abnormal CT findings in patients with signs of skull base fracture, visible signs of trauma above the clavicle, and suspected skull fracture were respectively 17.7, 14.8, and 14.6 times more than in patients without these findings.

The most common causes of head injuries were car accidents, falling from the height, street fights, falling to the ground, and trauma on face, consecutively, that were seen in 179 (44.7%), 97 (24.3%), 62 (15.5%), 49 (12.2%), and 13 (3.3%) patients, respectively. The majority of CTs were requested by emergency physicians, followed by neurosurgeons and ENT specialists who ordered 369 (92.3%), 14 (3.5%), and 8 (2%) CTs, respectively (Table 4). A comparison between emergency physicians and other specialists who ordered CT showed that emergency physicians ordered CT according to the indications list about 2.5 times more than other specialists (P = 0.01).

Table 4 shows the association between the specialty of physicians who ordered CT scans and existence of CT the indications. According to the indications list, the risk of inappropriate ordering of CT by ear, nose, and throat (ENT) specialists was about five times more than by emergency physicians. Sixty-one (15.2%) patients had at least one history of previous CT for MHT during the past 12 hours (Table 5). Among them, 26 (42.6%) patients did not have any indications of CT according to the indications list. In this study, 194 (48.5%) and 206 (51.5%) patients had and had not skull X-Ray for MHT prior to CT, respectively.

**Table 1.** Patients With Indications of Computed Tomography and Abnormal Computed Tomography Findings at the Same Time (n = 400) ^a^

Reason for CT^b^	Indications	Abnormal CT Reports ^c^
**Dangerous mechanism of injury**	180 (45)	45 (11.3)
Pedestrian or cyclist crash by a motor vehicle	92 (23)	22 (5.5)
Occupant ejected from a motor vehicle	9 (2.2)	8 (2)
Fall from a height > 1 m or five stairs	79 (19.8)	15 (3.8)
**Signs of skull base fracture**	88 (22)	40 (10)
Hemotympanum	2 (0.5)	0
Raccoon eyes	62 (15.5)	25 (6.3)
CSF Otorrhea/Rhinorrhea	10 (2.5)	5 (1.3)
Battle sign	5 (1.3)	2 (0.5)
Raccoon eyes & Battle sign	5 (1.3)	4 (1)
CSF Otorrhea/Rhinorrhea & Battle sign	2 (0.5)	2 (0.5)
All Signs	2 (0.5)	2 (0.5)
**Suspected skull fracture (open or depressed)**	70 (17.5)	34 (8.5)
**> 1 episode of vomiting**	40 (10)	13 (3.3)
**Age ≥ 65 years**	29 (7.2)	7 (1.8)
**Visible signs of trauma above the clavicle**	12 (3)	8 (2)
**Seizure**	9 (2.2)	1 (0.3)
**Amnesia for events > 30 min before incident**	7 (1.8)	0
**Coagulopathy**	0	0
**Patient with 2 indications simultaneously**	86 (21.5)	23 (5.7)
**Patient with 3 indications simultaneously**	31 (7.8)	20 (5)
**Patient with 4 indications simultaneously**	10 (2.5)	9 (2.2)
**Patient with 5 indications simultaneously**	1 (0.3)	1 (0.3)
**Patients with at least one indication**	253 (63.2)	54 (13.5)
**No indications**	147 (36.8)	0
**Total patients**	400 (100)	54 (13.5)

^a^ Data are presented as No. (%).

^b^ Abbreviations: CT, computed tomography; CSF, cerebrospinal fluid.

^c^ The declared percentages are from the total sample.

**Table 2.** Computed Tomography Findings According to Glasgow Coma Scale Score (n = 400)

GCS ^a^ score	GCS = 13-14		GCS = 15		Total No. (%)
CT report	No. (%) ^b^	% ^c^	No. (%)	%	
**Normal**	137 (34.3)	73.3	209 (52.2)	98.1	346 (86.5)
**Abnormal**	50 (12.5)	26.7	4 (1)	1.9	54 (13.5)
**Total**	187 (46.8)	100	213 (53.2)	100	400 (100)

^a^ Abbreviation: GCS, Glasgow coma scale; CT, computed tomography.

^b^ The declared percentages are from the total sample.

^c^ Percentage in GCS score group.

**Table 3.** Association of Indications With Abnormal Computed Tomography Findings (n = 400) ^a^

Indications	Odds Ratio	CI (95%)	P value ^b^
**Dangerous mechanism of injury**	7.8	3.70-16.50	< 0.001
**Signs of skull base fracture**	17.7	8.98-35.04	< 0.001
**Suspected skull fracture (open or depressed)**	14.6	7.63-28.10	< 0.001
**> 1 episode of vomiting**	3.7	1.80-7.83	< 0.001
**Age ≥ 65 years**	2.1	0.85-5.14	0.1
**Visible signs of trauma above the clavicle**	14.8	4.31-51.34	< 0.001
**Seizure**	0.8	0.10-6.50	0.83
**Amnesia for events > 30 min before incident**	-	-	0.292

^a^ Abbreviation: CI, confidence interval.

^b^ A P value less than 0.05 is statistically significant.

**Table 4.** The Association of Indication of Computed Tomography With Specialist Physicians Who Ordered the Imaging (n = 400) ^a^

Specialties	Indication		Odds Ratio	CI (95%)	P value ^b^
	Yes	No			
**Emergency physician**	240	129	1	-	-
**Neurosurgeon**	8	6	1.30	0.44-3.83	0.63
**ENT Specialist**	2	6	5.34	1.06-26.81	0.06
**General surgeon ** ^**c**^	3	0	-	-	0.30
**Neurologist ** ^**c**^	0	2	-	-	0.13
**Orthopedist ** ^**c**^	0	2	-	-	0.13
**Pediatrician ** ^**c**^	0	2	-	-	0.13
**Total **	253	147			

^a^ Abbreviation: CI, confidence interval.

^b^ A P value less than 0.05 is statistically significant.

^c^ Because the cell count in the table was zero, the calculation of odds ratio was not possible.

**Table 5.** History of Previous Computed Tomography During the Past Twelve Hours (n = 400)

History of Previous CT^a^	Indication			CT Result		
	Yes	No	Total	Normal	Abnormal	Total
**At least one **	35	26	61	47	14	61
**No history **	218	121	339	299	40	339
**Total**	253	147	400	346	54	400

^a^ Abbreviation: CT, computed tomography.

## 5. Discussion

The list of indications based on the world's most famous guidelines is nearly comprehensive and we tried to design it according to facilities existing in our country and recommendations of the specialists who ordered CT in public and private hospitals.

GCS scoring system is the standard method for evaluating the patient situation worldwide (19). It seems that this system is also widely used in Iran. In our study, approximately 37% of the patients did not have any indication of CT and no abnormal CT finding was reported in this group. Moreover, 13.5% of all patients undergone CT scan had abnormal CT reports. All patients with abnormal CT results had at least one of the indications.

Melnick et al. found that in the emergency department, about 10% to 35% of CT scans for patients with MHT were not indicated by evidence-based guidelines. They also concluded that effective implementation of the guiding principles could probably decrease ordering CT scans in MHT up to 35% and would cause a prominent decrease in health care costs. Their results were approximately similar to ours (9).

A study by the World Health Organization (WHO) showed that, the rate of abnormal CT results was approximately 5% in patients with GCS score of 15 and this rate may increase to 30% in patients with GCS score of 13 (20). Our findings demonstrated the percentages that were lower than the mentioned range that could be due to using different guidelines.

A recent study indicated that 13% of the patients with MHT (GCS score≥ 13) had abnormal findings in CT. In addition, patients younger than 65 years old with no obvious head injury, raccoon eyes, vomiting, and amnesia had normal CT results (21). Thus, the first part of their results approves our findings but with respect to the second part of their results, we attained different findings. A study by Iverson et al. demonstrated that the rate of abnormal CT results in MHT was about 16% and there was a statistically major link between the presence of intracranial abnormalities and lower GCS scores (22). In addition, a study by Ibanez et al. showed that patients with MHT had abnormal CT scans in 7.5% of the cases (23). Therefore, our findings demonstrated a rate between the results of the two studies and showed a statistically significant association between abnormal CT results and lower GCS scores.

Haydel et al. conducted a study in which about 6.5% of patients with MHT had an abnormal CT results. They also concluded that in MHT, CT could be performed only for patients with specific indications, which were similar to the mentioned risk factors in our study (10). In Turkey, Turedi et al. pointed out that in terms of abnormal CT findings, the difference between patients with GCS score of 13 to 14 and patients with GCS score of 15 was statistically significant (P < 0.0005). Moreover, the presence of two indications including vomiting and suspected skull fracture was significantly correlated with abnormal CT findings (respectively: OR, 4.61; 95% CI, 2.20-9.64; P = 0.0001; and OR, 3.46; 95% CI, 1.52-7.91; P = 0.0032) (24). Our findings were in line with the findings of the aforementioned study.

Two famous guidelines for ordering head CT after MHT (the Canadian CT Head Rules and the New Orleans Criteria) were compared in a study by Stiell et al. They demonstrated that the Canadian rules were more detailed; hence, using these rules caused a great decrease (25% to 50%) in the number of scans (11, 18). According to this study, the importance of attention to developing and implementing national guidelines regarding clinical status, resources, and the existing facilities access level is completely distinguished. Applying Clinical Practice Guidelines (CPGs) in the decision making process can help to improve the appropriate use of these diagnostic technologies. To our knowledge, there was no national guideline for referring patients with MHT to perform a CT scan up to the current study. A study in Iran on obstacles of CPGs development and implementation illustrated that the lack of an evidence-based health care system and a political macro support are the key barriers to produce and implement the CPGs (25). This study showed that a large number (37%) of patients with MHT that underwent CT scan in the emergency departments had no indication of CT; in addition, the majority (86.5%) of CT results were normal. The results of this study can be useful for the health specialists and policy makers in order to develop national guidelines toward appropriate use of health technologies and consider cost-effectiveness of these technologies. The use of best practice guidelines to manage the flow of patients who need a CT would help to improve proper access to this diagnostic tool and would decrease health care costs. Moreover, physicians’ decision making process would be easier when they order imaging on the basis of a national guideline.

Some limitations restricted the generalizability of the results of this study. The majority of patients were from urban area with insurance coverage and the results were based on the hospitals of Tehran city; therefore, other studies must be managed in other parts of the country to increase the generalizability of the results of this study. Moreover, it is possible that we had excluded some indications for which we could not make the consensus on the expert panel and this might have led to a slight overestimation of the inappropriate use of CT. Thus, results must be interpreted with cautions.
